# Supplementary material for: Patterns of Intron Gain and Loss in Fungi
Source: PLoS Biol. 2004 Nov 30;2(12):e422. doi: 10.1371/journal.pbio.0020422 (PMC532390; doi:10.1371/journal.pbio.0020422)
Supplement: Table S1 — Also available at http://genes.mit.edu/NielsenEtAl/. (4.3 MB ZIP). [file pbio.0020422.st001.zip › NielsenEtAl/html/230.html]

AN4549.1.NCU03173.1.MG03021.1.FG09541.1


```
 CLUSTAL W (1.82) Multiple Sequence Alignments - Introns Inserted


Sequence 1: NCU03173.1	419 aa
Sequence 2: MG03021.1	597 aa
Sequence 3: AN4549.1	407 aa
Sequence 4: FG09541.1	483 aa
Alignment Length: 629 aa
Number Identitical Residues: 108 aa
Alignment Score (without introns) 6088


MG03021.1 	MKTHSVFGRGTLGLLENGMHRRAPGKAVQHSFFLVNGRTSAASLLRKGDLASRQFSSTC~
NCU03173.1	---------------------------------------------MSGTFLGMQLRSGA~
FG09541.1 	--------------------------------------MQRFGVVGQCRFAARRLTQPR2
AN4549.1  	---------------------------------------------MRSQNLWRSLR-LV~
          	                                                      :     

MG03021.1 	PRTRKRSDVISG~SGKRKGGLNSEEFDVESFLRMGIDSSRDDRNDPTTKGPSPACHATSQ
NCU03173.1	DRCTFRAPYLAG2SNSRE------------------------------------------
FG09541.1 	TFHTSHTALSKP~KGSLG------------------------------------------
AN4549.1  	QRYSARSFLPAG~PSIRS------------------------------------------
          	     ::       .                                             

MG03021.1 	KLTERKYGSQSVGKSEDETRNMLALIEADNGRPSDEASFAFAFD2IDGVLLHQSEPLPGA
NCU03173.1	---ARKYNTKST--------------------PSEAPPFAFAFD2IDGVLLHVATPIPGA
FG09541.1 	-VNPNNFEEPTDLAR------------------KIFSEFAFAFD2IDGVLYQGRDRVDGA
AN4549.1  	---IRNLSSAAP----------------------RVPDFAFAFD2IDGVLLRSSTPIPGA
          	    .:    :                         . ****** ***** :    : **

MG03021.1 	TDALRFLQANKIPFILLTNGGGKHEHERVAELSAKLGVPLTTG----NFVQSHTPFS---
NCU03173.1	PEALKFLNDNDIPFILLTNGGGKHETERVKDLSQKLGVELTTD----NFVQSHTPFRQLV
FG09541.1 	DKVIKMLRSNGIRYVFLTNGGCVPESKKEESLQERLQIPKHEDAIKGRMILSHTPMSGWS
AN4549.1  	AESLALLKKEGIPFILLTNGGGKHETERVAEISEKLQVPLDAD----VIIQSHSPFAEMV
          	 . : :*. : * :::*****   * ::  .:. :* :    .     :: **:*:    

MG03021.1 	-GLNR---FMDKNILVTGSDAAKSREIAEA~YGFTKVITPADILMANPTIWPFDPLLESV
NCU03173.1	DGPDS---LREKTILVTGANAEKCRLIAEE~YGFRNVVTPADILKAHPEVFPFDPLLDTV
FG09541.1 	EDIKN-----DGTILITGSHPEKARQIALD2YGFKRVVTPADILAECGDVFPFEHIEGEI
AN4549.1  	RGTESQPALENKRVLVVGGDGDGCRAVAEK~YGFKNVVTPGDIFMANPSIWPFSKGFSDY
          	 . . ..:  :  :*:.*..   .* :*   *** .*:**.**:     ::**.      

MG03021.1 	YASTARPLPQPIYNKNPYMKE-WPKRLSRDNLQIHAVFIFNDPRDWALDIQIITDLLLSH
NCU03173.1	YTATARPLPRPIFTPGSGM------RLS-DALKIDAMFVFNDPRDWAYDIQLIVDLLLSQ
FG09541.1 	NG---KPVPLPDGKRIPLLKDPYSTNIPANALKIDHIFIWNDPRDWSVDIQLIHDLLISH
AN4549.1  	YKTFTRPLPHKGEPGDPTMG-----------LKIDAIFVFNDPRDWALDAHIIIDLLLSS
          	  : ::*:*       . :            *:*. :*::******: * ::* ***:* 

MG03021.1 	SGVLGTYSAANS----VNGKWPEDKNPWLFYSNSDLLWASKHPLPRMGQGAFQAALAGVW
NCU03173.1	QGYVGTYSPKNGDAALPSCGWQQDGQPPLYFSNADLLWSTGFHLPRFGQGAFQAAVAGVW
FG09541.1 	QGYLGTVSNLNGNEKLPNNGWQQDGQPGLWISNLDLLWKTNYPVNRFGTGAFMEALKGVW
AN4549.1  	QGVLGTISDKNGRANLPNRGYQQDGQPPLYFSNPDLWWAAAYSLPRLGQGGFREALEGMW
          	.* :** *  *.     .  : :* :* *: ** ** * : . : *:* *.*  *: *:*

MG03021.1 	ADISDT----LGVRGSGVRSGPRCTRFGKPIVQTYQFAERVLMEHRRQLLGEEVANHR--
NCU03173.1	RRLTNG----HELQRR---------VIGKPYSETYQFAERVLTTHRHEVLRRRGHHEPG-
FG09541.1 	STTTNG----KELEYS---------ALGKPSNHTYRYAHERLLQYYHDMACNRGQSPGHD
AN4549.1  	SAITGGSNKGVELQKT---------VIGKPSQGTYEFAEKQLLRNRSRAF---AFETTR-
          	   :. :...  :.            :***   **.:*.. *                  

MG03021.1 	-----HLRNVYMVGDNPASDIAGANAFKGKSRIGTDWWGVLVKTGVFNPDIS----MPLQ
NCU03173.1	-----TLKSVYMVGDNPESDIAGANDFH--SEAGTEWCSILVRTGVWSPEHAGEKALQGR
FG09541.1 	KSKYHPLRRVYMIGDNPESDIRGASEFE--AEDGTEWVPILVRTGVWRQTATE---KEPR
AN4549.1  	-----PLRNVYMVGDNPESDICGANSYR--SAHNSEWHSILVRTGVYMGGEP-------A
          	      *: ***:**** *** **. :.  :  .::*  :**:***:    .        

MG03021.1 	HKPKMIVDDVFEAVKWALGREQWRGP-PLVMATREAAVGESHEGLVENNVIRNDGENMAK
NCU03173.1	FKPKVIVDDAREAVRWALKREGWTGP-AL-------------------------------
FG09541.1 	YKPVVIVDDVVDALVWALNNEGIKADREWVLSALSHTKGYVKLPPLEDGVHNGDLEVGAK
AN4549.1  	WTPKTIADNVQKAVEWGLQHSNWQTK----------------------------------
          	 .*  *.*:. .*: *.* ..                                       

MG03021.1 	GDLLQGDGNVVEGKIIKDGEEVVVEGETIKEGK
NCU03173.1	---------------------------------
FG09541.1 	---YPSELEATEAKL------------------
AN4549.1  	---------------------------------
          	
```
